# “COVID-19 in Trials and Tribulations” Project: A Self-Regulation-Based Support Response for Confined Families through Social Networks

**DOI:** 10.3390/ijerph19031910

**Published:** 2022-02-08

**Authors:** Armanda Pereira, Paula Magalhães, Sara Teixeira, José Carlos Núñez, Daniela Rosendo, Sandra Mesquita, Raquel Azevedo, Joana Araújo Martins, Sonia Fuentes, Pedro Rosário

**Affiliations:** 1 Department of Education and Psychology, School of Human and Social Sciences, University of Trás-os-Montes and Alto Douro, 5000-801 Vila Real, Portugal; 2 Psychology Research Center, School of Psychology, University of Minho, 4710-057 Braga, Portugal; pcsmagalhaes@gmail.com (P.M.); sarateixeira.psi@gmail.com (S.T.); danielapatriciamr@gmail.com (D.R.); sandrampmesquita@gmail.com (S.M.); raquel.azevedo.mota@gmail.com (R.A.); a78129@alunos.uminho.pt (J.A.M.); prosario@psi.uminho.pt (P.R.); 3 Faculty of Psychology, University of Oviedo, 33003 Oviedo, Spain; jcarlosn@uniovi.es; 4 Facultad de Educación y Ciencias Sociales, Universidad Central de Chile, Santiago 530-598, Chile; sofu2028@gmail.com

**Keywords:** COVID-19, social networks, elementary school children, narrative-based program, self-regulation

## Abstract

The COVID-19 pandemic has placed today’s society in an unprecedented scenario. During Portugal’s first home confinement period (March–July 2020), the online-based “COVID-19 in Trials and Tribulations” project was implemented to support families with school-aged children. The project was grounded on the self-regulation framework and delivered through Facebook^®^ and Instagram^®^ pages. Being responsive to ongoing developments of the pandemic, activities were conveyed in two phases. Phase 1 occurred during lockdown (school was suspended). Phase 2 occurred while students were enrolled in at-distance (online) school. The present study aimed to examine the reach of the project, while examining the content and format of delivery that generated the most engagement among the users (4500 Facebook^®^ effective followers; 1200 Instagram^®^ effective followers) during the confinement period. Results showed that, at the individual page level, Facebook^®^ had higher reach indicators compared to Instagram^®^, except for video. At the Facebook^®^ post level, followers and users showed more engagement with the page prior to the at-distance schooling phase; however, videos still generated engagement (*p* = 0.002). Both the post type (*p* < 0.01) and frequency (*p* < 0.001) of publication were suggested to be good predictors of engagement. The information gathered will help design and inform future interventions that may be implemented as new lockdowns are set in place.

## 1. Introduction

As of 18 March 2020, Portugal declared a state of emergency due to the spreading of the new coronavirus disease in the 2019 (COVID-19) pandemic [1]. Like many other countries [2], the state of emergency ordered, among its several measures, school closures, home confinement and, for many, remote work [1]. Given the initial circumstance, the Portuguese government authorities’ announcement of school closures was not accompanied by an expected return date, and few guidelines were provided on how schools should organize to continue promoting learning opportunities to their students [3]. Although the swift and acute measures were progressively relaxed in May as the country transitioned from a state of emergency to a calamity status [4], most students did not return to school. High school students returned physically to school on 18 May 2020 [3], but students from the remaining school levels remained at home with access to television-school or online synchronous sessions (i.e., online classes delivered by the teacher through online platforms to which students were expected to attend from an electronic device at home).

Among the many challenges created by the COVID-19 pandemic, the continuity of distance learning joined the list of major priorities that governments around the world needed to address [5]. Following school closures, several countries elected to implement digital tools providing educational support to children in attempts to mitigate the negative impacts related to the COVID-19 outbreak [6]. Although necessary, this decision was accompanied by concerns, especially for parents, regarding children’s ability to self-regulate their learning from home, as well as parents’ capability to support it [7].

This new state of affairs pushed families to alter their routines and discover ways of managing their newfound roles and responsibilities [8]. Parents converted their homes into office spaces and classrooms and found themselves interchanging between roles as professionals, schoolteachers, housekeepers, and caregivers.

In such a situation, being able to reorganize one’s life, establish goals, make plans to attain them, and master unusual tasks relies largely on the person’s capacity to self-regulate their behaviors [9]. The detrimental consequences of these unplanned changes in family structure, along with long periods of social isolation may expose strengths and frailties of individual and collective resources. In fact, public health disasters, such as the coronavirus pandemic, seem to significantly impact on mental health [10,11], namely through the disturbance of people’s usual activities, due to the implementation of measures to control the virus’s spread (e.g., quarantine) [12]. One way to dampen the impact of the stress-inducing event on mental health might be through the regularization and maintenance (or replacement) of daily routines [13].

Amid this scenario, the project “COVID-19 in Trials and Tribulations” (for an extended description, see Section 2.2.) was devised to support Portuguese families with school-aged children during the home confinement and social isolation period. The aim of “COVID-19 in Trials and Tribulations” was to help regularize secondary routines (e.g., leisure activities and exercising) through a diverse range of suggestions (e.g., activities to carry out at home, stories, and quotes to reflect critically on) available online on Facebook^®^ (facebook.com/covid19emsarilhos, accessed on 18 January 2022) and Instagram^®^ (instagram.com/covid19_em_sarilhos, accessed on 18 January 2022) pages. The project activities aimed to help families cope with the challenges posed by school closures and home confinement (e.g., promote children’s agency toward schoolwork, set physical activity routines likely to prevent sedentary behaviors, foster children’s interests and responsibilities towards family daily routines). This decision was aligned with the results of a previous meta-analysis, suggesting positive outcomes when social networking sites were utilized in the learning context, particularly in the informational and emotional support domains [14].

### 1.1. Theoretical Framework

“COVID-19 in Trials and Tribulations” Online built on Yellow’s Trials and Tribulations intervention program. Yellow’s Trials and Tribulations [15] was a story tool, centered on the intervention program developed by the same authors. This story told the adventures of the colors of the rainbow searching for their friend Yellow, who was lost in the woods. As in any adventure, there were challenges and difficulties to overcome, and for the colors to succeed in their purpose of finding their friend, they had to first equip themselves with self-regulatory competencies. The goal of this program was to train children ages 7 to 10 with a set of transversal self-regulation competencies and promote children’s autonomy and agency (i.e., intentional action towards a goal, mobilizing the necessary resources, and influencing the environment) [15,16,17]. The plot provided the scenario for children to acquire declarative (e.g., what is planning), procedural (e.g., how is planning executed), and conditional (e.g., when and why is planning necessary) self-regulated learning (SRL) processes and strategies. The program was anchored in the social cognitive framework [18], particularly grounded in Zimmerman’s SRL Model [19,20]. This model supported the PLEE (Planning, Execution, and Evaluation) model, as follows [21]: (a) the planning phase describes students’ efforts to define their learning goals and select the learning strategies best suited to help them attain their goals; (b) the execution phase is displayed when students implement and monitor the execution of the plan established in the previous; and finally, (c) the evaluation phase occurs when students analyze the outcomes of the execution phase against the initial plan. In particular, by training and developing SRL competencies, students are encouraged to assume an agential role in their learning process, i.e., to take responsibility and control over their educational paths. Furthermore, by employing the SRL strategies to control their own behavior, cognition, motivation, and environment, students are more likely to maintain and improve their academic performance and engagement with their learning processes [22]. Extensive research has shown the positive impacts and effectiveness of this intervention in promoting self-regulatory skills, self-efficacy, and behavioral and cognitive classroom engagement [8,17,23,24]. 

Students who self-regulate their learning are likely to develop self-generated thoughts, emotions, and behaviors intentionally driven towards attaining self-set learning goals [25]. Therefore, providing opportunities for families to promote these competencies with their children (through the presentation of activities with a self-regulatory rationale) may help them cope with difficulties, particularly in this pandemic scenario.

### 1.2. The Present Study

In Portugal, by March of 2020, all in-person activities were suspended due to the state of emergency precipitated by the pandemic. Acknowledging the potential of social networks to deliver educational content [26], we intended to understand the extent to which users engaged with educational and recreational content delivered by social media pages. 

Analyzing Portuguese families’ access to digital devices and the internet, data from INE (Instituto Nacional de Estatística—Statistics Portugal) indicated that about 80% of Portuguese families had internet access, mostly for entertainment purposes (games, social network pages, and movies), at the time of the study. Moreover, about 80% of the Portuguese population participated in social networking pages, and about 76% of this participation was via smartphone devices [27,28,29]. Moreover, Portuguese users dedicated about 96 min per day to social networking pages [30]. Specifically, Facebook^®^ was the most highly used page by the Portuguese population (92.2%), followed by WhatsApp^®^ (80.3%) and then Instagram^®^ as the third most popular (73.3%) [30]. Grounded on these metrics, *COVID-19 in Trials and Tribulations* Online was developed simultaneously through Facebook^®^ (facebook.com/covid19emsarilhos, accessed on 18 January 2022) and Instagram^®^ (instagram.com/covid19_em_sarilhos, accessed on 18 January 2022) pages. Considering the data from Portuguese social network users, the largest group of Facebook^®^ users was between the ages of 25 and 44, and the largest group of Instagram^®^ users was between the ages of 25 and 34 [31]. We did not select WhatsApp^®^ because of its chat format (i.e., the app functions with conversation windows one on one or with a group of people). Therefore, the publications would not have been available to everybody, only to the users previously added to the group. In addition, we would have needed permission to access and add people’s phone numbers to the group, per the app’s functionality.

In the present study, we examined the extent to which Facebook^®^ and Instagram^®^ were useful tools to reach families while delivering education-related content during a pandemic period. Furthermore, we were interested to learn the publication format (e.g., video, picture) with which users were most engaged. Therefore, the present study aimed to examine the reach of the project and to ascertain which content and format of delivery generated more engagement among the users. Briefly, we set the following hypotheses: (1) while delivering educational and recreational content, both social networks would be strong tools to reach families during a pandemic period, and (2) video content would be the publication format that generated more engagement from families. 

Note: we had no expectation about the direction of the hypothesis and of the results regarding participants’ engagement on T1 and T2 because when we started the project, it was not possible to anticipate how the virus would behave and the implications attached (e.g., school closure or dates for reopening of face-to-face classes).

## 2. Materials and Methods

### 2.1. Target Population

“COVID-19 in Trials and Tribulations” was devised to reach a wide range of families with school-aged children (elementary school children, i.e., between 6 and 11 years old). The recruitment process took place in two phases: (1) families from schools across the country who were enrolled from the beginning of the school year (September 2019) in the Yellow’s Trials and Tribulations intervention program to promote self-regulated learning skills were invited to participate in the COVID-19 in Trials and Tribulations project (a continuation of the previous one, now in an online format) and (2), in addition, after the first pool of families were engaged, other people started following the pages (snowball effect). To comply with legal requests, the login for the “COVID-19 in Trials and Tribulations” social networking pages was made formally by parents or legal guardians; no exclusion criteria was set for enrollment. After the login on the “COVID-19 in Trials and Tribulations” social networking pages, the first resource that parents or legal guardians had access to was an introductory video about the “COVID-19 in Trials and Tribulations” project. The age range of parents or legal guardians following the social networking pages was congruent with national data about the median age of parents or legal guardians of school-aged children in Portuguese families (25–44) [32].

### 2.2. COVID-19 in Trials and Tribulations

Acknowledging the pandemic restrictions as well as the literature [33], Facebook^®^ and Instagram^®^ were elected as the means to deliver the intervention (The following supporting information can be consulted at: https://www.instagram.com/covid19_em_sarilhos/ (accessed on 18 January 2022) and https://www.facebook.com/covid19emsarilhos/ (accessed on 18 January 2022)). Two purposes underlaid this intervention: (1) to deliver educational and recreational content likely to help families regulate routines at home, specifically using activities for their children to be done autonomously or with adults, and for the entire family, and (2) to reach the maximum number of families in confinement with school-aged children.

All content was developed by the research team together with key specialists in diverse areas, such as educational and social fields. These partnerships allowed the research team to check whether the content built was adjusted to (a) the educational needs of the families in confinement (e.g., physical exercise, healthy eating routines, study tips) and (b) developmental acquisitions and academic competencies (e.g., reading and writing) within the children’s age range. For instance, all the content created was supported by visual aids, i.e., images and/or videos. Moreover, “COVID-19 in Trials and Tribulations” developed educational content suitable for children with disabilities, as well as for raising awareness about children with special needs, to ensure that all children could participate. These specific materials were created in collaboration with a cerebral palsy rehabilitation center from northern Portugal.

**Figure 1:**
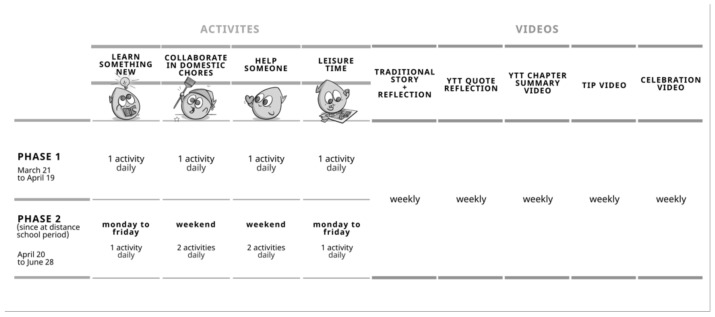
Educational content delivery scheme of “COVID-19 in Trials and Tribulations” Facebook^®^ and Instagram^®^ pages.

Educational content was delivered daily via “COVID-19 in Trials and Tribulations” Facebook^®^ and Instagram^®^ pages. This content was made up of two forms: (1) weekly videos and (2) daily activities (see Figure 1). The weekly videos included five types of content as follows: (i) A traditional weekly story enacted by the members of the research team (e.g., “Ali Babá and the 40 thieves”; and “Three Little Pigs”), followed by a reflection embedding SRL strategies. (ii) A weekly reflection on a particular quote from Yellow’s Trials and Tribulations. (iii) A weekly summary video about one of the chapters from Yellow’s Trials and Tribulations. (iv) A weekly tip from educational experts; these tips, delivered by specialists in the fields of psychology and education, aimed to help parents, guardians, and children develop coping strategies. Topics included aspects relevant to the lockdown context, such as study methods, screen time, healthy eating habits, and anxiety management skills. Finally, (v) a weekly celebration; at the end of each week, a video was posted in which all “COVID-19 in Trials and Tribulations” team members celebrated the end of one more week in home confinement (see Figure 1).

Daily activities were organized into four categories: (i) “learn something new” (e.g., virtually visit the Louvre), (ii) “collaborate in domestic chores” (e.g., make the bed), (iii) “help someone” (e.g., writing a thank-you letter to the medical staff), and (iv) “leisure time” (e.g., playing a family board game). These categories followed the recommendations of the United Nations Education, Scientific and Cultural Organization (UNESCO) [34], which were recently updated to address the impacts of school closures on students’ learning processes and mental and physical health (see Figure 1).

The category “learn something new” included activities that provided children with opportunities to continue their learning, i.e., bringing school to children’s homes. This was achieved by stressing the importance of adapting their study rhythm to the new reality while maintaining study-related activities. The content of the activities could be of a curricular (e.g., learning a new drawing technique) or extracurricular (e.g., virtually visiting museums) nature. They could also be self-regulated learning strategies (e.g., learning to take notes [35]).

The category “collaborate in domestic chores” included activities promoting children’s awareness of the necessity of collaborating with household members at home. This was achieved by stressing the importance of maintaining a positive home environment, which could be challenging due to the families being restricted to the same physical space while performing different activities and duties (e.g., telework, distance learning). Thus, the activities were designed to promote organizational routines (e.g., clearing the bedroom) and create opportunities to do handicraft activities as a family (e.g., building a keychain).

The category “help someone” included activities promoting communication between children and their peers, grandparents, and other family members despite the physical distance and activities encouraging humanization during the lockdown period. This was achieved by providing opportunities for children to help others (e.g., make tutorial calls with friends who have difficulties in math exercises) or to be grateful for something or someone (e.g., write a letter of gratitude to a teacher/nurse/fireman for his/her dedication).

**Figure 2:**
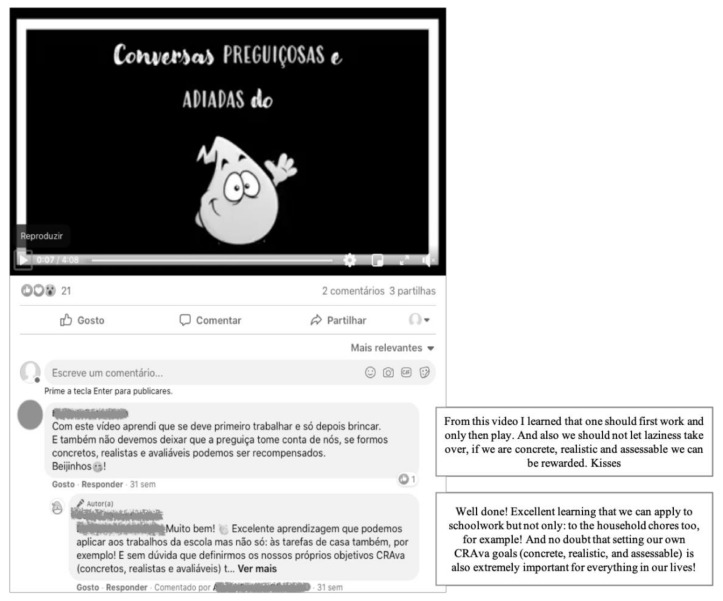
Facebook^®^ post—example of the category “learn something new” with the title “Yellow’s lazy and postponed talks”.

**Figure 3:**
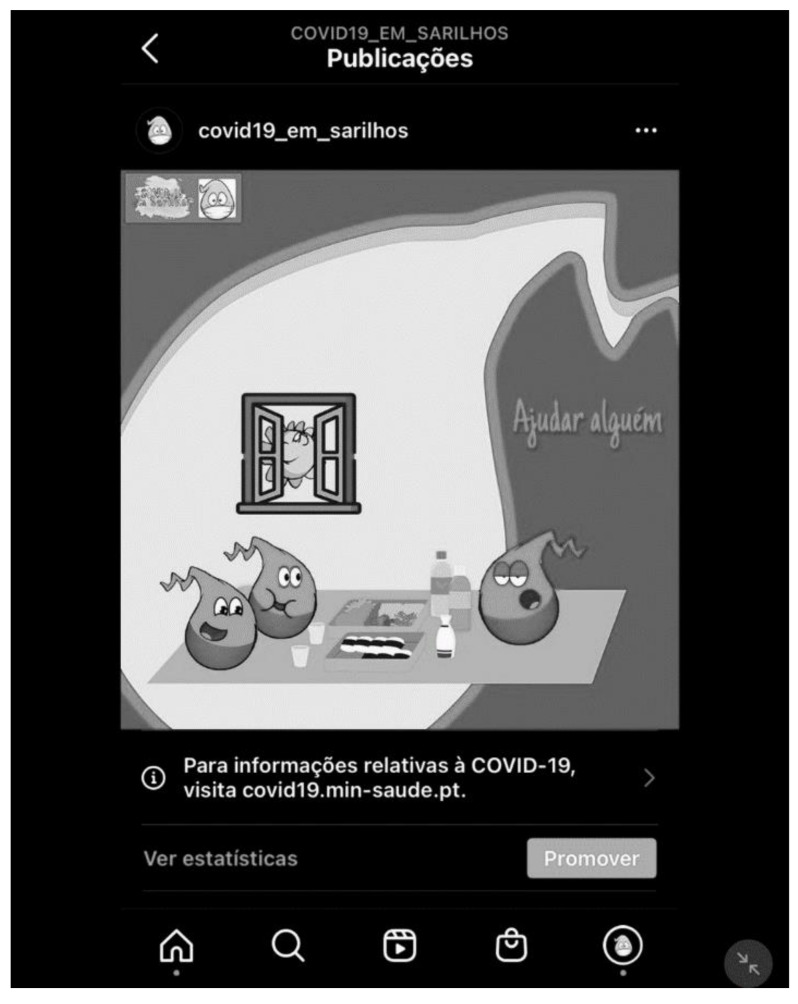
Instagram^®^ post—example of the category “help someone” with the title “Indoors picnic with the family”.

The last category, “leisure time”, included activities minimizing the impacts of sedentary behavior (e.g., screen time) associated with home confinement, and noncurricular activities to be shared with the family. This was achieved by highlighting the importance of combatting sedentarism to prevent the development of detrimental physical and mental health conditions. Thus, the activities were designed to provide opportunities for physical exercise (e.g., burpees training), non-screen leisure activities (e.g., board games), and (on and off-screen) family leisure activities (e.g., movie night). Figure 2 and Figure 3 show two examples of publications on the Facebook^®^ and Instagram^®^ COVID-19 Trials and Tribulations pages, respectively.

Acknowledging the role played by the self-regulation processes (e.g., goal planning) in helping individuals cope with novel and stressful situations (e.g., self and task management [9]) or with engaging successfully in online learning tasks [36,37], all the set activities were grounded on the social cognitive self-regulation framework [24,38].

The implementation of the daily activities was comprised of two phases. Phase 1 occurred from 21 March 2020 until 19 April 2020. During this phase, there were four daily publications, one per category (i.e., “Learn something new”, “Collaborate in domestic chores”, “Help someone”, and “Leisure time”). During those 4 weeks, the country was on lockdown, and all school-related activities were suspended. For data analysis purposes, Phase 1 corresponded to Time 1 (T1) of data collection. Phase 2 occurred from 20 April 2020, until 28 June 2020. During this phase (10 weeks), many Portuguese students were enrolled in at-distance schools (e.g., teleschool, online classes). To address this increase in school-related work and the associated busier schedules, an adjustment was made to the number of activities delivered on a daily basis. During phase 2, there were only two publications daily (“Learn something new” and “Leisure time”) from Monday to Friday, and four publications per day (adding back in “Collaborate in domestic chores” and “Help someone”) on Saturdays and Sundays. For data analysis purposes, Phase 2 corresponded to Time 2 (T2) of data collection.

### 2.3. Data Collection and Measures

Data was collected using the Facebook^®^ and Instagram^®^ Insight tool. These tools allowed access to detailed data during the lifetime of the page. Demographic information (e.g., gender, age, distribution, and location of the followers) was collected to characterize the followers of the “COVID-19 in Trials and Tribulations” pages. Other lifetime metrics were collected for 14 weeks, from the day the pages were launched on 21 March 2020, until the publications were discontinued on 28 June 2020 (the end of the school year). The metrics collected between 21 March 2020, and 19 April 2020, corresponded to T1 of data collection (pre-at-distance schooling). The metrics collected between 20 April 2020, and 28 June 2020, correspond to T2 of data collection (at-distance schooling).

#### 2.3.1. Facebook^®^ and Instagram^®^ Page-Level Information

Regarding the “COVID-19 in Trials and Tribulations” Facebook^®^ and Instagram^®^ pages, the following weekly metrics were selected for analysis: (i) Likes/Followers, i.e., the number of unique users who have liked/followed the page; (ii) Unlikes/Unfollowers, i.e., the number of unique users who had unliked/unfollowed the page; (iii) Total Engagement, i.e., the number of unique users who had engaged with the page, including any clicks or stories created; (iv) Total Reach, i.e., the number of unique users who had content from or related to the page appearing in their timelines; (v) Total Viral Reach, i.e., the number of people who had any content from or about the page entering their screen with social information attached (as a form of organic distribution, social information was displayed when a person’s friend interacted with the page); (vi) Total Consumption, i.e., the number of clicks on any of the page’s content—stories generated without clicking on the page’s content (e.g., liking the page on the Timeline) were not included; (vii) Total Video Views, i.e., the number of times a video was viewed due to organic reach; (viii) Total Video Repeat, i.e., the number of times the video was seen outside of the first play. Please note that not all these metrics are available for the Instagram^®^ page (e.g., post-level information).

#### 2.3.2. Facebook^®^ Post Level Information

Regarding the “COVID-19 in Trials and Tribulations” Facebook^®^ post-level information, engagement metrics (the sum of likes, comments, and shares) were collected for several variables: category (i.e., “Learn something new”, “Collaborate in domestic chores”, “Help someone”, “Leisure time”, “Tips”, “Quote”, “Story”, *Yellow Trials and Tribulations* (YTT) Story, and “Celebration”), post type (video or photo), and Phase (Phase 1 corresponded to T1, when school activities were suspended [pre-at distance schooling], and Phase 2 corresponded to T2, when at-distance schooling was implemented).

### 2.4. Data Analysis

The dataset was exported from Facebook^®^ and Instagram^®^ using Insight tools, and the data was analyzed using SPSS^®^ 24 [39]. Descriptive statistics were conducted to describe the page-level variables. Regarding the Facebook^®^ post level analysis, the number of likes, shares, and comments were summed to create the engagement variable [40]. Engagement data (dependent variable) was gathered per week and organized per category (e.g., “learn something new”) and post type (video or photo) (independent variables). This data was analyzed considering the phase of implementation (T1 or T2). An ANOVA was conducted to test differences in engagement, considering variables such as category, post type, and phase of implementation. Lastly, a regression analysis was conducted to test for the predictors of engagement.

## 3. Results

The “COVID-19 in Trials and Tribulations” Facebook^®^ accumulated 4500 effective followers, 84% of whom were female, with an age range of 35–44. The top five cities with the most garnered followers on the Facebook^®^ page were Braga, Porto, Guimarães, Lisboa, and Bragança, and the top five countries from which followers joined were Portugal, Brazil, Switzerland, France, and Spain. The “COVID-19 in Trials and Tribulations” Instagram^®^ accumulated 1200 effective followers, 84% of whom were female, with an age range of 25–44. The top five cities that garnered followers on the Instagram^®^ page were Braga, Porto, Lisboa, Guimarães, and Bragança, and the top five countries with most followers were Portugal, Brazil, Switzerland, Spain, and the United Kingdom.

The results were reported as follows: (i) a general descriptive analysis of the Facebook^®^ and Instagram^®^ pages’ metrics; (ii) for the Facebook^®^ page, an analysis of the participants’ engagement as a function of the activity category; and lastly, (iii) for the Facebook^®^ page, a data analysis comparing our page’s performance against other similar pages.

### 3.1. Page-Level Description

Table 1 displays descriptive statistics regarding the metrics for the Facebook^®^ and Instagram^®^ pages. The Facebook^®^ page, compared with Instagram^®^, had more reach in the majority of the indicators—except for “Total Video Views”. Both pages had a very small number of unlikes.

### 3.2. Facebook^®^ Post Level Analysis

Table 2 displays the engagement data (likes, comments, and shares) in the *COVID-19 in Trials and Tribulations* Facebook^®^ posts. The mean engagement is displayed per category (e.g., “Learn something new”) as a function of the post type (video or photo) for T1 and T2 (Phase 1, pre-at-distance schooling, and Phase 2, during at-distance schooling, respectively).

**Figure 4:**
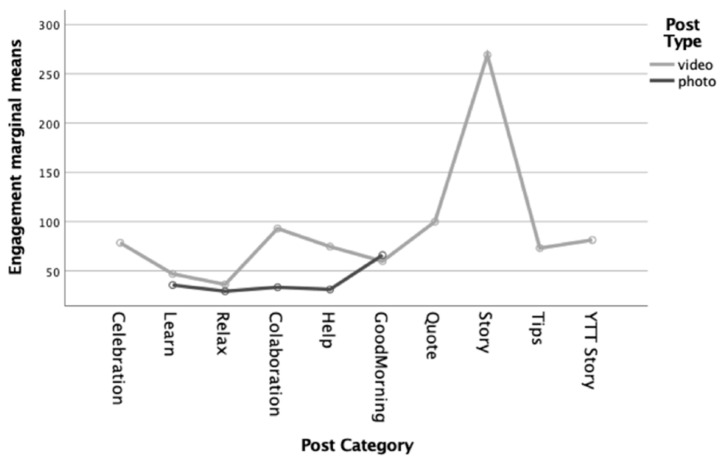
Mean engagement level regarding post type (video or photo) and content categories (Celebration, Learn, Relax, Collaboration, Help, Good Morning, Quote, Story, Tips and Yellow Trial and Tribulation story [YTT]—for more detailed information, please see Section 2.2.).

Considering time (T1 and T2), univariate analysis results showed significant differences in engagement level regarding category *F* (1, 27) = 33.83, *p* < 0.001, η^2^*_p_* = 3.64 and post type *F* (1, 27) = 9.58, *p* = 0.002, η^2^*_p_* = 0.23. Overall, results showed that engagement was higher during T1, when children had no school activities, compared with T2, after school had restarted. Furthermore, videos, when compared with photos, particularly stories, generated more engagement from users (Figure 4). Engagement levels for all post categories decreased in T2. Despite the pronounced decrease, the story category persisted as the category in which users were most engaged. Congruently, engagement levels for the post types also decreased in T2. Despite the acute decrease, the video type remained the post type with which users were most engaged. A post hoc Tukey test showed that the category types “Good Morning”, “Celebration”, “YTT Story”, “Tips”, and “Quote” within the subgroup differed with marginal significance at *p* = 0.07.

Lastly, a multiple linear regression was calculated to predict engagement based on post type (i.e., video or photo) and frequency of the post types (i.e., weekly or daily). A significant regression equation was found (*F* (2438) = 47.86, *p* < 0.001) explaining 18% (*R* = 0.42) of the engagement level within the Facebook^®^ content. Both post type and frequency of post types seemed to predict engagement level (β = −0.24, *t* = −5.21, *p* < 0.01; β = 0.28, *t* = 6.11, *p* < 0.001, respectively). However, the frequency of post types was the predictor with major statistical significance, meaning that weekly posts better predicted the engagement level within the page contents.

## 4. Discussion

The COVID-19 pandemic brought unprecedented changes to families all around the globe. Among Portuguese families, particularly those with school-aged children, it called for a complete rearrangement of family routines to accommodate pandemic telework, house chores, and at-distance schooling [8]. Within this scenario, the “COVID-19 in Trials and Tribulations” project was devised to support families during the first home confinement and social isolation period, namely, to aid the management and regulation of new routines at home. The present study aimed to examine the reach of the “COVID-19 in Trials and Tribulations” project and examine which content and delivery mode generated maximal engagement among users. Facebook^®^ and Instagram^®^ were the social networks selected to host the project. From a global data standpoint, these seemed the most relevant and appropriate tools to reach our target population (i.e., families with school-aged children) [30].

“COVID-19 in Trials and Tribulations” pages reached followers across the Portuguese territory, as well as countries overseas (e.g., Brazil, France). Furthermore, considering the results at the individual page level, Facebook^®^ had higher reach indicators compared to Instagram^®^, with one exception: video views. This result suggested that Instagram^®^ could better host this specific educational content (e.g., stories and educational tips in videos). 

Regarding the Facebook^®^ post level results, followers and users showed more engagement with the page’s content before the at-distance schooling phase started. Recent exploratory studies have systematized the challenges accompanying at-distance schooling, such as children’s lack of routines and the absence of strategies and methods to help children learn more autonomously from home. These challenges prompted the need for extra assistance from parents or other family members [41].

The overlap of settings and tasks (i.e., school and work) at home may have shortened the time and availability to engage with other activities, such as social networking pages. However, both post category (e.g., “learning something new”) and post type (e.g., video) showed a high effect size (>0.14). The post category, jointly with the post type, seemed to contribute to maintaining engagement. However, we still noted that categories that were delivered in video format seemed to generate higher engagement across time. Specifically, results showed that videos still generated engagement during the at-distance schooling phase, especially story videos. This was clearly illustrated through the feedback about stories sent via private messages from parents’ personal accounts (e.g., “Dear team of the “COVID-19 in Trials and Tribulations”, my daughter is an unconditional fan of your stories. She is 3 years old and repeatedly asks to see the stories that you post. Thank you and congratulations for the amazing work!”—Follower, mother of a 3-year-old girl). These findings were consistent with Facebook^®^ moderator recommendations, in which videos were defined as vivid content that contributed to increased engagement on social networking pages [33]. 

The home confinement context required self-regulatory skills to control the management of new home-school routines and cognitive flexibility to adjust plans to meet unpredicted external demands (e.g., telework, at-distance schooling, and ongoing restrictive measures imposed by the authorities). The contents of “COVID-19 in Trials and Tribulations” were devised to assist in the development and/or maintenance of these routines through activities encompassing self-regulatory skills. In addition, to address the needs of Portuguese families during the home confinement period, post type and frequency planning were responsive to changes related to the governmental measures defining the lockdown conditions. In this sense, it became critical to understand what type and frequency of posts best predicted families’ engagement with the page’s contents. The results suggested that both post type and publication frequency were good predictors of families’ engagement with the “COVID-19 in Trials and Tribulations” Facebook^®^ page. Notwithstanding, the weekly frequency of posts stood out as the best predictor. This could be explained by the fact that the weekly publications were disseminated via video format. This result further highlighted the relevance of considering audiovisual material in the design of educational content for intervention programs using social networking applications. 

### 4.1. Implications for Practice: Lessons Learned

From the very first moments of the new reality of lockdown, families were confronted with the need to establish new routines and adjust familiar and personal dynamics. Thus, through Phase 1 of the “COVID-19 in Trials and Tribulations”, the project’s team intentionally directed efforts to deliver content daily to help families who were establishing new routines and coping with the challenges and hardships of the lockdown. For Phase 2, characterized by at-distance school activities, the “COVID-19 in Trials and Tribulations” team adjusted all content and post-publication frequency to fit this new governmentally imposed scenario.

The project’s responsiveness to families’ ongoing needs (adaptation from no school activities to an at-distance school scheme) was key for families’ adapting to the new schedule. The changes to the mode and frequency of project activities were responsive to parents and educators’ feedback throughout the program (e.g., post comments, private messages to the team), especially in the transition period, i.e., from Phase 1 to Phase 2. During this period, we closely followed the OECD [5] recommendations for establishing and maintaining communication with families. These guidelines were critical in attempts to minimize the families’ mental health struggles associated with the COVID-19 lockdown. Moreover, selecting social networks as the vehicle to deliver this project ensured that the communication with families and children who were enrolled in projects to promote SRL prior to the COVID-19 pandemic was maintained. In addition, social networks can be accessed from accessible and commonly used technological devices (e.g., smartphone), increasing the spectrum of families reached (e.g., families with low income). Moreover, in Portugal, the majority of the municipalities’ administrations throughout the pandemic ensured internet access to resident families with school-age children and/or adolescents. 

This project, as well as the management of the system of deliveries and communication with followers and users, built upon the SRL rationale. Throughout the project, we monitored the flow of communication to learn of the families’ necessities so that we could adapt the activities, post timings, and overall schedule to their needs. Importantly, we aimed to promote families’ safety by helping them feel supported at home when taking care of their children in confinement. We believe that the program’s responsiveness to the needs of the target population was the key factor for their engagement on our social network pages. The use of social networks to enable online interventions in an educational context has been increasingly utilized, dating to long before the lockdown [42,43]. Consistent with prior findings, data on the “COVID-19 in Trials and Tribulations” suggested the feasibility of Facebook^®^ and Instagram^®^ platforms to deliver educational content in an emergency state period. We believe that factors such as the consistency, intentionality, and congruence of the content published in the “COVID-19 in Trials and Tribulations” pages may help explain the promising results. Still, we would like to acknowledge some limitations to the current research.

### 4.2. Limitations and Future Studies

Despite being preliminary, the results suggested positive engagement with both pages’ educational content. Still, this study had limitations that warrant acknowledgment. One of the major limitations of our study was the unfeasibility of verifying that the followers’ ages corresponded to the age of the real users of the page. In other words, it was not possible to confirm if children were using parents’ mobile phones and/or accounts to access the pages’ content, or if parents/legal guardians were showing the pages’ content to their children. Our target population was confined to families with school-aged children, who may or may not have accessed the pages through one single account. 

In addition, we were unable to ensure that our followers were all families with school-aged children. Aware of these limitations, our paper focused on the user’s engagement with the content of the pages. Regardless of the lack of control over family-related data, several users shared photos and videos displaying activities developed with their children (e.g., baking a cake with the family using the PLEE), which was a positive indicator. 

The lockdown was unexpected for everyone. Thus, there were limited opportunities to gather robust data on the efficacy of the intervention. Nevertheless, despite these limitations, this study demonstrated the feasibility of using these online platforms to convey educational content and promote transversal self-regulation competencies. Thus, future research could consider assessing the impact of similar interventions in a non-pandemic scenario, to promote mental health and overall well-being among families.

## 5. Conclusions

The COVID-19 pandemic created an unprecedented scenario for everyone, including families of school-aged children. “COVID-19 in Trials and Tribulations” project was devised to provide support for families with school-aged children during the first home confinement period in Portugal. The project was delivered through Facebook^®^ and Instagram^®^ pages, and the results showed that social networking pages could be a relevant tool to deliver educational content, aiming to support at-distance. We believe this project to have had merits beyond the pandemic context in which it was developed; in the future, researchers could consider examining the relationship between engagement with the implementation format of at-distance interventions and the development of self-regulation competencies and overall well-being.

The purpose of the current paper was to present an online response to the families’ educational needs and understand the families’ engagement with the educational content created. This program was created in alignment with the “Yellow’s Trials and Tribulations” story tool, which stated: “There is a way, there is always a way. Those who don’t give up will succeed”.

## Figures and Tables

**Table 1:** Page-level information, reporting the means (M), standard deviations (SD), minimum, and maximum for the metrics selected along the 14 weeks of the intervention.

	M (SD)	Min	Max
Facebook^®^ N = 14 weeks			
Likes	328.79 (629.70)	17	2362
Unlikes	11.50 (18.75)	2	73
Total Engagement	9787.50 (4356.40)	5558	17,648
Total Reach	83,888.50 (39,232.46)	44,660	158,228
Total Viral Reach	55,899.86 (25,090.13)	30,590	118,531
Total Consumption	14,090.36 (3743.14)	10,325	21,848
Total Video Views	58,837.07 (20,933.13)	32,271	104,065
Total Video Repeat	27,525.71 (5988.34)	11,789	34,357
Instagram^®^ N = 14 weeks			
Followers	1132 (135.60)	836	1247
Unfollowers	7.43 (10.46)	0	38
Total Engagement	1613.50 (2172.14)	881	9147
Total Consumption	543.29 (338.62)	188	1279
Total Video Views	33,966.43 (32,608.60)	471	81,255

**Table 2:** Facebook^®^ mean engagement (likes, comments, and shares) per category as a function of the type of post (video or photo). Total number of posts in each category is also displayed. T1 corresponds to Phase 1 of the implementation, and T2 corresponds to Phase 2.

			Post Type			
	Total nr. of Posts		Video		Photo	
	T1	T2	Mean Engagement T1	Mean Engagement T2	Mean Engagement T1	Mean Engagement T2
Category: Daily						
LSN ^a^	31	54	62.50	31.32	39.43	31.74
CDC ^b^	29	47	93.00	--	35.18	31.60
HS ^c^	32	48	114.60	34.60	32.85	29.50
LT ^d^	28	54	42.50	29.93	31.96	26.67
GM ^e^	0	39	--	59.79	--	66.00
Tips	0	2	--	57.00	--	--
Category: Weekly						
Quote	4	10	129.00	70.90	--	--
Story	3	18	444.33	98.00	--	--
Tips	10	8	72.40	90.00	--	--
YTT Story	4	6	112.00	50.67	--	--
Celebration	4	10	95.25	62.70	--	--

Note: ^a^ LSN—Learn Something New; ^b^ CDC—Collaborate in Domestic Chores; ^c^ HS—Help Someone; ^d^ LT—Leisure Time; ^e^ GM—Good Morning.
